# MEMS Acoustic Sensors: Charting the Path from Research to Real-World Applications

**DOI:** 10.3390/mi16010043

**Published:** 2024-12-30

**Authors:** Qingyi Wang, Yang Zhang, Sizhe Cheng, Xianyang Wang, Shengjun Wu, Xufeng Liu

**Affiliations:** 1 School of Basic Medicine, Air Force Medical University, Xi’an 710032, China; wangqingyi@fmmu.edu.cn; 2 School of Military Medical Psychology, Air Force Medical University, Xi’an 710032, China; chengsizhe0527@fmmu.edu.cn (S.C.); wangxianyang_1999@163.com (X.W.); wushj@fmmu.edu.cn (S.W.); 3 School of Biomedical Engineering, Air Force Medical University, Xi’an 710032, China; yangzhang@fmmu.edu.cn

**Keywords:** MEMS, acoustic sensor, working principles, performance, application

## Abstract

MEMS acoustic sensors are a type of physical quantity sensor based on MEMS manufacturing technology for detecting sound waves. They utilize various sensitive structures such as thin films, cantilever beams, or cilia to collect acoustic energy, and use certain transduction principles to read out the generated strain, thereby obtaining the targeted acoustic signal’s information, such as its intensity, direction, and distribution. Due to their advantages in miniaturization, low power consumption, high precision, high consistency, high repeatability, high reliability, and ease of integration, MEMS acoustic sensors are widely applied in many areas, such as consumer electronics, industrial perception, military equipment, and health monitoring. Through different sensing mechanisms, they can be used to detect sound energy density, acoustic pressure distribution, and sound wave direction. This article focuses on piezoelectric, piezoresistive, capacitive, and optical MEMS acoustic sensors, showcasing their development in recent years, as well as innovations in their structure, process, and design methods. Then, this review compares the performance of devices with similar working principles. MEMS acoustic sensors have been increasingly widely applied in various fields, including traditional advantage areas such as microphones, stethoscopes, hydrophones, and ultrasound imaging, and cutting-edge fields such as biomedical wearable and implantable devices.

## 1. Introduction

Acoustic systems are characterized by their use of sound waves as carriers of information and energy. They are specifically applied in measuring sound wave intensity, reproducing sound wave waveforms, and determining sound pressure frequency. Acoustic sensors [1], designed for monitoring acoustic systems, are commonly found nowadays in various fields, including household appliances [2], industrial equipment, military operations [3,4], health maintenance [5], disease diagnosis [6,7], and scientific research. Cases of acoustic sensor utilization are also abundant, precisely in detecting low-frequency noise [8] through nanofiber piezoelectric materials, monitoring mechanical processing [9], alarm-based acoustic devices for intruder detection [10], and making hydrophones for underwater sound wave detection [11]. With their increased application in various fields, the demand for better performance of the devices is more evident [12]. In order to obtain and monitor acoustic information of targets more accurately and reliably, acoustic sensors need to have better sensitivity, bandwidth, noise resolution, signal-to-noise ratios, etc., as well as be modified in terms of size, robustness, integration, and other properties.

Microelectromechanical systems, commonly known as MEMS, comprise an ingenious technology that has overarching applications in multiple domains. The rapid evolution of MEMS research has been instrumental in the Industrial Revolution 4.0, which heavily relies on the Internet of Things (IoT) for data accumulation in developing smart living environments. Given the market trend in recent decades and future technology outlooks, MEMS is expected to be a steadily growing, multibillion-dollar industry [13,14]. MEMS acoustic sensors, in combination of traditional acoustic sensing and MEMS technology, have been applied in microphones, hydrophones, biosensors, etc. MEMS acoustic sensors are evolving with improved miniaturization, high performance, multimode, intelligence, integration, adaptability, flexibility, etc. Today, MEMS acoustic sensors can meet performance requirements with their high sensitivity [15], high resolution [16,17], and high directionality [18,19] in their applications. Due to the differences in application scope and sensing principles, it is difficult to find a consistent standard to measure or compare all devices. But through some representative examples, we can see the current state-of-the-art performance parameter level. Taking sensitivity as an example, a silicon-cantilever-based MEMS acoustic sensor with fiber-optic Fabry–Perot interferometric readout method can realize an ultrahigh sensitivity of 1.753 μm/Pa at 1 kHz and 28.75 μm/Pa at the resonance frequency [20]; an active fiber Fabry–Pérot microcavity design can reach a sensitivity of 2.6 V/Pa at the resonance frequency [21]. As for resolution, AlN- and d_33_-mode-based piezoelectric MEMS acoustic sensors can acquire an enhanced signal-to-noise ratio of 67 dB, which is 7–30 dB higher than state-of-the-art results for the same types of devices, and a noise resolution of 2 μV/√Hz, which is the lowest result as compared to the state-of-the-art results for the same types of devices [22]. As a comparison, the sensitivity of commercial traditional acoustic sensors typically ranges from a few to tens of mV/Pa and relies on circuit post-processing to achieve a better signal-to-noise ratio and noise resolution. For example, the well-known RCA 44 vintage ribbon microphone is side-addressed with a bidirectional polar pattern and has a sensitivity rating of 2.5 mV/Pa. Another vintage classic ribbon microphone, the Coles 4038, is side-addressed with a bidirectional polar pattern and has a sensitivity rating of 0.6 mV/Pa.

**Figure 1:**
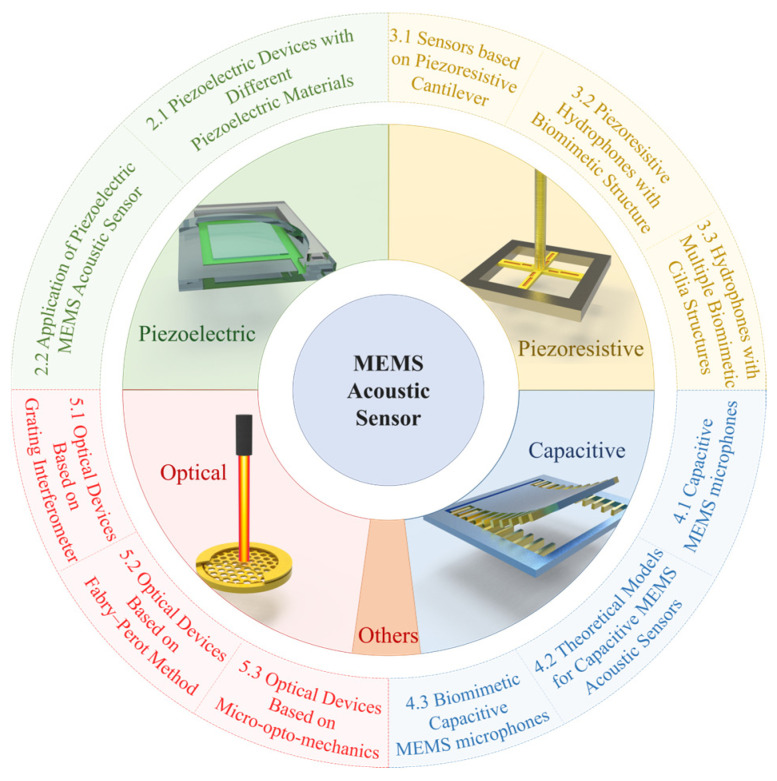
Classification of MEMS acoustic sensors based on different working principles.

Due to the introduction of silicon-based microstructures and microfabrication processes, MEMS acoustic sensors have been shown to have unique features over traditional ones. For example, they can withstand high-reflow soldering temperatures, integrate with CMOS technology and other audio circuits, and have improved noise cancellation and good RF and EMI suppression. The products using this technology have also demonstrated many advantages in various applications. The common working principles of MEMS acoustic sensors include piezoelectric [23], piezoresistive [24], capacitive [25], and optical [26] ones, as shown in Figure 1. Therefore, in this article, we focus on these four categories of devices, detailing recent advancements in MEMS acoustic sensor development and showcasing innovations in structure, processes, and design methods. Additionally, we compare the performance of devices with similar operating principles. Due to space limitations, we are unable to include all types of device forms. Other sensor types, such as electromagnetic acoustic sensors and electret acoustic sensors, have also made significant progress in recent years and have been widely applied, e.g., chip-based radar sensors, MEMS thermo-acoustic sensors [27], and electret microphones [28].

## 2. Piezoelectric Method

**Figure 2:**
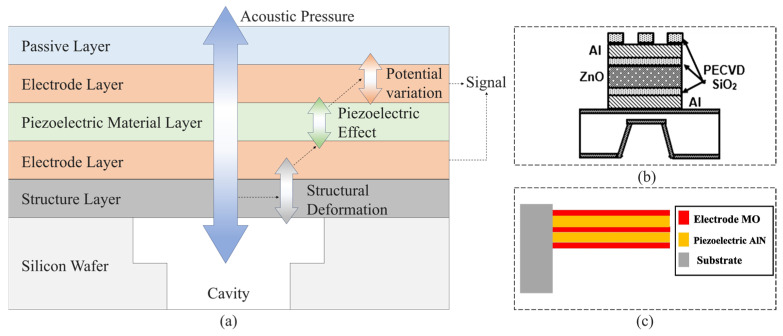
Piezoelectric MEMS acoustic sensors. (**a**) Basic working principle and typical multilayer structure of piezoelectric MEMS acoustic sensors. (**b**) A ZnO MEMS acoustic sensor with air cavity [29]. (**c**) Multilayer cantilever design of a piezoelectric MEMS microphone, with AlN as piezoelectric material and MO as an electrode material [30].

The typical designs of piezoelectric MEMS acoustic sensors include the piezoelectric cantilever beam or boundary supporting film, with conductive layers coated on two sides (Figure 2a). These sensitive structures’ deformation under the input acoustic waves can lead to an electrical signal output through the conductive layers. The behavior of a piezoelectric acoustic sensor (PAS) can be evaluated with the help of orientation, thickness, and selection of piezoelectric material and diaphragm structure resonant frequency [29], as shown in Figure 2b. Ahmed Fawzy et al. [30] developed a simulation platform, MEMS microphone optimizer platform (MMOP), to design high-performance cantilever piezoelectric MEMS microphones with sensitivity estimation, as shown in Figure 2c. It can predict wide-ranged issues key to the successful design of a MEMS microphone, such as the optimum values of piezoelectric material thickness, electrode material thickness, and the length of a cantilever. MMOP also enables the direct simulation of sensitivity from the input parameters of the designed model.

### 2.1. Piezoelectric MEMS Acoustic Sensors Based on Different Piezoelectric Materials

Piezoelectric materials such as AlN, ZnO, PZT, and LiNbO_3_ have been used in MEMS acoustic sensor development. These piezoelectric materials are used to construct various thin films for accurate capture of sound waves. Piezoelectric thin film (PTF) materials present unique properties, including high sensitivity, wide dynamic range, wide displacement, and low power consumption [31]. Non ferroelectric piezoelectric materials, like ZnO and AlN, possess a crystal structure called wurtzite and are used at high frequencies for acoustic applications. PZT films have a high percentage of lead, which is hazardous to the environment and humans due to its toxic nature. Jacek Baborowski’s team from the Swedish Federal Institute of Technology studied PZT thin-film piezoelectric ultrasonic sensors with a film thickness of 1 μm [32]. PZT thick-film sensors have received significant attention, due to the minimal thickness of piezoelectric thin films and notable surface effects. For example, Nanyang Technological University in Singapore has developed a micro ultrasonic sensor based on PZT thick film, of 7 μm [33].

The ever-growing applications of PZT thin films to sensing devices have given birth to a variety of microsensors [33]. Thin-film PZT offers many unique advantages due to its high electromechanical coupling factors, permittivity, piezoelectric stress constants, and the DC-bias electric field dependence of these properties [34]. Much progress has been made in its deposition and processing technologies. The sol–gel method can be used to deposit thick PZT film for multilayered bimorph pMUT transducers [35]. Yamashita et al. [36] fabricated a piezoelectric ultrasonic microsensor with a PZT capacitor on a buckled diaphragm and investigated the deposition condition of the sol–gel derived PZT films from the viewpoint of high mechano-electrical conversion efficiency. PZT diaphragms fabricated by sol–gel PZT thin film processes can exhibit improved generated power density and be used as PZT MEMS acoustic energy harvesters [37]. The structural design in PZT devices, like patterned transducer arrays and ridges among the elements, can effectively limit the cross coupling between elements, reduce the parasitic capacitance, and increase the coupling coefficient [35]. Dry etching techniques like ion beam etching are widely used for patterning PZT thin films. Zhu et al. [38] proposed a PZT MEMS acoustic sensor with Pt/PZT/TiO_2_/SiO_2_ structure for microphone and microspeaker applications. The maximum sensitivity is around 11 mV/Pa, and its resonance frequency is around 44 kHz. Chen et al. [39] proposed a PZT MEMS acoustic sensor with two-stage amplification that consists of an asymmetric gapped cantilever and a charge amplifier, and achieved a high sensitivity of 9.2 V/g at frequencies of less than 1000 Hz, making it suitable for use in monitoring weak physiological signals, including heart and lung sounds. PZT ceramics, with excellent piezoelectric performance, can be used to form MEMS acoustic sensors with high practicality. PZT ceramic interdigital sensors can accurately collect vibration signals on the surface and further analyze extra loads, allowing it to be used for circumstances like non-destructive structural testing and gas sensing [40]. The introduction of flexible materials enables it to have better conformability and mass sensitivity as an acoustic flexural plate wave sensor [41]. In particular, MEMS piezoelectric energy harvesters with PZT ceramics can be used as fully implantable cochlear implants to stimulate the auditory nerve inside the cochlea, therefore eliminating the use of microphones, sound processors, batteries, and transmitters in conventional cochlear implants [42]. The signals generated by multiple cantilevers with varying resonance frequencies within the hearing band can stimulate the different corresponding sections of the auditory nerve [43]. In addition to wave energy sensing, a ceramic PZT with a high-piezoelectric constant can also be applied as a piezoelectric MEMS speaker with a high sound pressure level [44].

ZnO is a highly piezoelectric material with excellent dielectric properties, direct band gap (3.27 eV), high activation energy, favorable piezoelectric coefficient, and coupling coefficient [45]. An acoustic sensor based on PZT has low sensitivity in comparison to a ZnO-based acoustic sensor [46]. The relative dielectric constant of PZT is almost 100 times greater than that of ZnO, which affects the capacitance of a device with the same dimensions. AlN has high phase velocity, with a chemically stable and hard stage, but controlling the texture and stoichiometry of the film is more challenging compared to ZnO film deposition [47].

**Figure 3:**
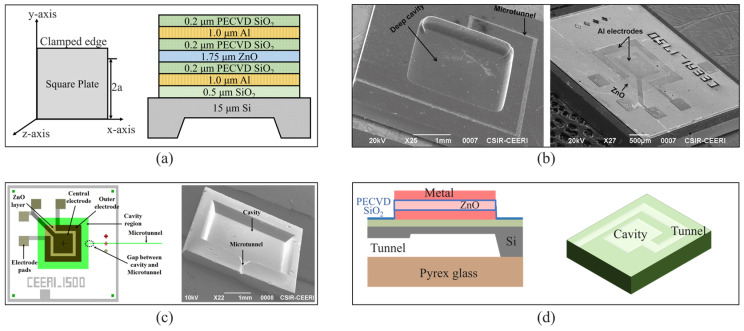
Piezoelectric MEMS acoustic sensor based on ZnO film. (**a**) ZnO based structure for development of MEMS acoustic sensor [29]. (**b**–**d**) The cavity structure with microtunnel design, which relates to the atmosphere, as a replacement of the traditional acoustic holes. (**b**) The fabricated cavity and metal electrode structure of ZnO MEMS acoustic sensor [48]. (**c**) A ZnO MEMS acoustic sensor for aeroacoustic measurements [50]. (**d**) A MEMS acoustic sensor with microtunnel for high SPL measurement, and with less risk of microtunnel blockages [51].

Kumar et al. [29,48] reported a series of piezoelectric ZnO-based acoustic sensors with a large dynamic range, 100–180 dB sound pressure level (SPL), and large bandwidth (12~22 kHz) for audio and aeroacoustic applications. The highest sensitivity is 80 μV/Pa (Figure 3a,b). The annealing temperature optimization plays an important role in determining the fabrication process parameters and generation of voltage corresponding to the pressure applied on an acoustic sensor. There is a directly proportional relation between the crystallinity of ZnO films and annealing temperature. The surface reactions and species mobility are affected by the annealing temperature [31]. Kumar et al. also investigated the influence of the annealing process on a ZnO MEMS acoustic sensor’s performance [49]. The sensor has a square-shaped diaphragm with a side length of 1750 μm. The fabricated structure has been annealed at different temperatures, and the maximum piezoelectricity of the annealed ZnO film is at 400 °C. Ali et al. [50] demonstrated a ZnO MEMS acoustic sensor for aeroacoustic measurements. The low cut-off frequency, bandwidth, and flat band sensitivity of the sensor have been found to be 48 Hz, 54 kHz, and 130 μV/Pa, respectively (Figure 3c). Prasad et al. [51] proposed a MEMS acoustic sensor with a microtunnel for high SPL measurement, and presented an approach to remove microtunnel blockages during sensor fabrication. The sensor structure consists of a piezoelectric ZnO film deposited between two Al electrodes on a thin silicon diaphragm (Figure 3d). The microtunnel relates the cavity to the atmosphere, as a replacement of the traditional acoustic holes made in diaphragm, back plate, or glass substrate for acoustic pressure compensation, protecting the device from any microcrack generation or fracture and improving the reliability as well as fabrication yield of the device.

AlN is advantageous in sensing applications, including microphones, due to lower dielectric and acoustic losses, which result in a high signal-to-noise ratio (SNR) and voltage output. It has properties such as low temperature deposition (under 500 °C), compatibility with ICs, good thermal stability, and high acoustic velocity [52]. AlN has a dielectric constant 100 times less than that of PZT, with excellent chemical stability, a very high breakdown field, non-toxic material, and non ferroelectric material that does not need poling [31].

**Figure 4:**
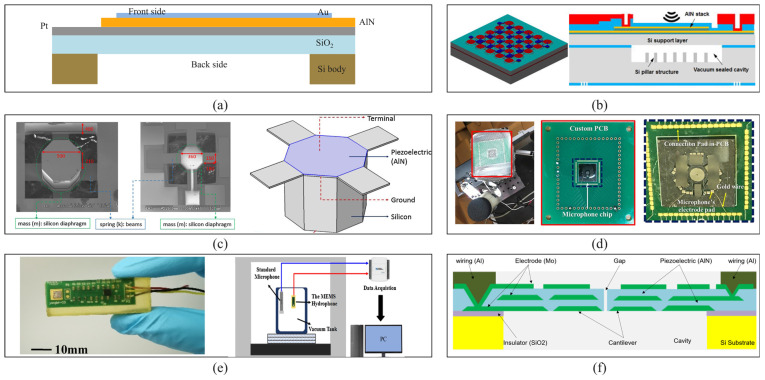
Piezoelectric MEMS acoustic sensor based on AlN. (**a**) A AlN pMUT based on the compatibility characteristics between AlN and CMOS processes [53]. (**b**) AlN MEMS acoustic sensor aiming for ultra low working frequency [54]. (**c**) AlN MEMS acoustic sensor with ultra-thin silicon substrate, and different structures for low and high working frequency [56]. (**d**) AlN MEMS acoustic sensor with enhanced SNR (67.03 dB at 1 kHz) [22]. (**e**) AlN MEMS hydrophone with high sensitivity (−178 dB, re. 1 V/μPa) and low noise density (52.6 dB@100 Hz, re. μPa/√Hz) [58]. (**f**) AlN MEMS wideband (10 Hz to more than 10 kHz) acoustic sensor coated by organic film (elastic polyurethane) [59].

Przybyla et al. [53] proposed an AlN piezoelectric micro mechanical ultrasonic transducer based on the compatibility characteristics between AlN and CMOS processes, and pointed out that compared to piezoelectric ceramics, the application of AlN materials can improve the signal-to-noise ratio (Figure 4a). The MEMS ultra-low frequency hydrophone using aluminum nitride piezoelectric material proposed by Xu et al. [54] has a working frequency of 10–100 Hz and a sound pressure sensitivity of −182 dB, achieving high sound pressure sensitivity for MEMS piezoelectric hydrophones (Figure 4b). The ultra-thin silicon substrate aluminum nitride Lamb wave piezoelectric resonator proposed by Li et al. [55] from the Chinese Academy of Sciences can achieve high-precision detection of trace substances. Their research shows that Lamb wave sensors using aluminum nitride have the advantages of high chemical stability, compatibility with CMOS, and high temperature resistance. Kabir et al. [56] proposed piezoelectric MEMS sensors for detecting elastic waves released by active flaws (Figure 4c). The designed sensors operate in plate flexural mode or rigid body mode driven by the vibration of a silicon diaphragm on which the piezoelectric layer is deposited. This sensor has highly narrow band frequency response and superior sensitivity/size compared to bulk AE sensors.

A major factor that determines the performance of an acoustic sensor is the SNR, which also indicates the smallest sound pressure that can be detected by the sensor [57]. Rahaman et al. [22] developed a MEMS piezoelectric acoustic sensor with 7–30 dB higher SNR as compared to the state-of-the-art of PAS. This AlN PAS uses d_33_ transducer mode with a nominal electrode spacing of 20 μm. At 1 kHz frequency, the measured SNR is found to be 67.03 dB, which varies from 70 dB to 85 dB in the bandwidth, the equivalent input-referred noise (EIN) is 26.97 dB SPL, and the A-weighted noise is 27.23 dBA, which is the lowest noise ever analytically and experimentally reported in the state-of-the-art of PAS (Figure 4d). Yang et al. [58] presented a micromachined hydrophone with high sensitivity and low noise density. The hydrophone is composed of a 10-by-10 piezoelectric AlN membrane array and low-noise amplification circuit, and is packaged by an acoustic transparent material. A new pouring method is applied to improve the acoustic performance of the matching layer. The experimental results show that the packaged MEMS hydrophone achieves an acoustic sensitivity of −178 dB (re. 1 V/μPa), a bandwidth from 100 Hz to 1600 Hz, and an equivalent noise density of 52.6 dB at 100 Hz (re. μPa/√Hz) (Figure 4e). Kuchiji et al. [59] developed a MEMS wideband acoustic sensor with cantilever array structure, using AlN and polyurethane as the piezoelectric material and organic film, respectively. By covering the gap between the cantilever and highly elastic polyurethane, they managed to increase the allowable amount of warpage and improve the sensitivity in the low-frequency region (below 1 kHz). Filling the gap between the cantilevers with polyurethane is expected to increase the acoustic resistance and decrease the cutoff frequency of the acoustic high-pass filter (Figure 4f).

Another piezoelectric material that has been widely applied for MEMS acoustic sensors is ScAlN. ScAlN film has a large-value electromechanical coupling coefficient and high longitudinal wave velocity, even when deposited by simple sputtering methods at low temperatures of around 200 °C [60]. Zhou et al. [61] presented a ScAlN high-frequency MEMS vector hydrophone composed of four groups of sensing cell arrays. It acquires large operational bandwidth for acoustic pressure gradient measurement underwater, and has promising potential in biological detections. It operates in the frequency range of 20 kHz to 160 kHz and is specially designed to monitor dolphin calls. The measured maximum reception sensitivity is −175 dB (re. 1 V/μ Pa) in the dipole receive mode at 160 kHz and −169 ± 2 dB (re. 1 V/μ Pa) in the omnidirectional receive mode in the operating bandwidth, respectively. Hu et al. [62] proposed a ScAlN-based piezoelectric MEMS microphone with sector-connected cantilevers for wearable medical detection and commercial acoustic products. The output of this piezoelectric MEMS microphones assembly is amplified and filtered from 100 Hz to 20 kHz, and the measured sensitivity, minimum detectable pressure, resolution, total harmonic distortion, and signal-to-noise ratio are −37.6 dB (re. 1 V/Pa), 40 dB SPL, 40 dB SPL, 0.997%, and 54.2 dB at 1 kHz, respectively. Liu et al. [63] developed a wearable ScAlN MEMS acoustic sensor for identification in harsh noisy environments. A hexagonal structural design and ScAlN piezoelectric film are used to improve the sensitivity of the acoustic sensor. In addition, the proposed sensor also has the advantages of ultra-wideband (10 Hz–20 kHz), ultra-flat frequency response (±0.5 dB).

Overall, PZT, ZnO and AlN are the most commonly used piezoelectric materials in MEMS acoustic sensors. Table 1 gives the performance comparison of several recent MEMS acoustic sensors. On this basis, researchers have proposed numerous innovations, which includes utilizing air channels to enhance the reliability of multi-layer film structures, as well as integrating multiple structures to adapt to high-frequency and low-frequency working environments, and ultimately achieve improvements in performance indicators such as sensitivity, operating bandwidth, and noise resolution.

### 2.2. Application of Piezoelectric MEMS Acoustic Sensors

Microphones, as a widely used consumer electronic device, are the most important application scenario for acoustic sensors, and the same applies to piezoelectric MEMS acoustic sensors. MEMS microphones have become increasingly popular due to their many advantages over traditional microphones. They offer lower power consumption, smaller size, and higher sensitivity, making them an excellent choice for a wide range of applications [64]. Prasad et al. [51] developed a MEMS microphone with a microtunnel structure for high sound pressure level measurement. Zhu et al. [38] utilized the piezoelectric effect and converse piezoelectric effect of PZT film to form a micro-acoustic device for both microphones and microspeakers. In addition to microphones in consumer electronics, piezoelectric MEMS microphones are also involved in more specific fields, for example, on the exterior of aircraft during flight tests to enable characterization of turbulent boundary layers. To meet this demand, the device should be compact in size, passive, and have a linear response at large sound pressure [48], which is in line with the advantages of piezoelectric MEMS acoustic sensors. Kumar et al. [48] reported a MEMS microphone with square-shaped diaphragm along with microtunnel for high sound pressure level measurement in launching vehicles and aircraft. In addition to the audible frequency range, piezoelectric MEMS microphones can also achieve a wider working frequency range. Kuchiji et al. [59] developed an AlN MEMS microphone with a piezoelectric cantilever array structure covered with a polyurethane organic film, which helped to improve its sensitivity in the low-frequency region and increase the resonance frequency. As the demand for smaller, more efficient audio devices continues to grow, MEMS microphones are likely to play an increasingly important role in the audio and sensor industries [64].

**Figure 5:**
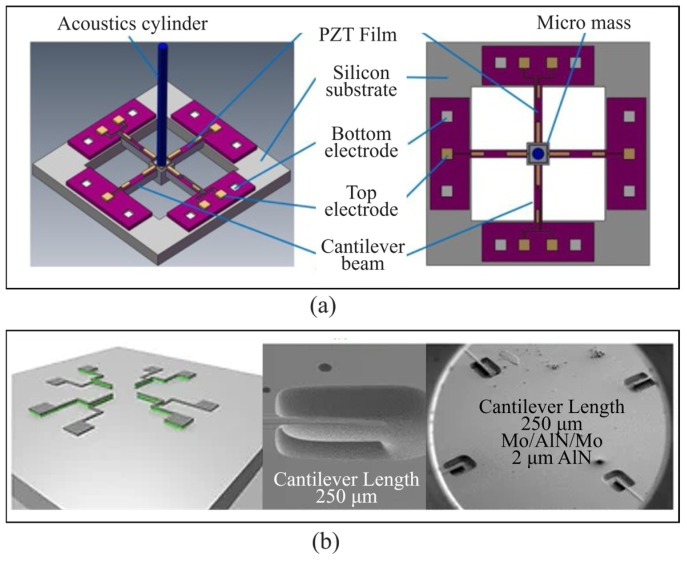
Piezoelectric MEMS hydrophone. (**a**) A face to face, cross-configuration of four cantilevers design [67]. (**b**) Single cantilever beam design [68].

The development of piezoelectric MEMS acoustic sensors in recent years has expanded beyond traditional microphone applications to include specialized devices such as hydrophones, as well as wearable acoustic sensors that are applied for human health condition detection. Jinghui Xu et al. [65] developed a 10 × 10 element arrayed MEMS hydrophone device with a Mo-AlN-Mo sandwiched piezoelectric layer. It achieves an acoustic sensitivity of −180 dB (re. 1 V/μPa), a bandwidth from 10 Hz to 8 kHz, and a noise resolution of around 60 dB (re. 1 μPa/√Hz) at 1 kHz. Licheng Jia et al. [66] proposed a novel sensing cell design of honeycomb architecture based on an AlN-on-cavity silicon-on-insulator (CSOI) platform, for achieving large acoustic pressure sensitivity of −178 dB (re. 1 V/μ Pa) with a maximum nonlinearity of 0.1%, high fill-factor, and a noise resolution of 58.7 dB (re. 1 μPa/√Hz). Shuzheng Shi et al. [67] presented a PZT film-based vector hydrophone that consists of four cantilevers and an acoustic columnar cilium fixed on the center micro mass (Figure 5a). This passive MEMS piezoelectric hydrophone has the characteristics of receiving sensitivity of −189.3 dB at 920 Hz (0 dB = 1 V/μPa) and a typical “8” shaped directional pattern. Basit Abdul et al. [68] reported a hydrophone with four AlN thin-film cantilevers in a cross configuration. The 200 nm thick molybdenum electrode thin layers in the Mo-AlN-Mo structure add a stress gradient through cantilever thickness, leading to out-of-plane cantilever bending, showing maximum sensitivity of up to −163 dB (Figure 5b). Table 2 shows the performance comparison of several recent piezoelectric MEMS hydrophones.

**Figure 6:**
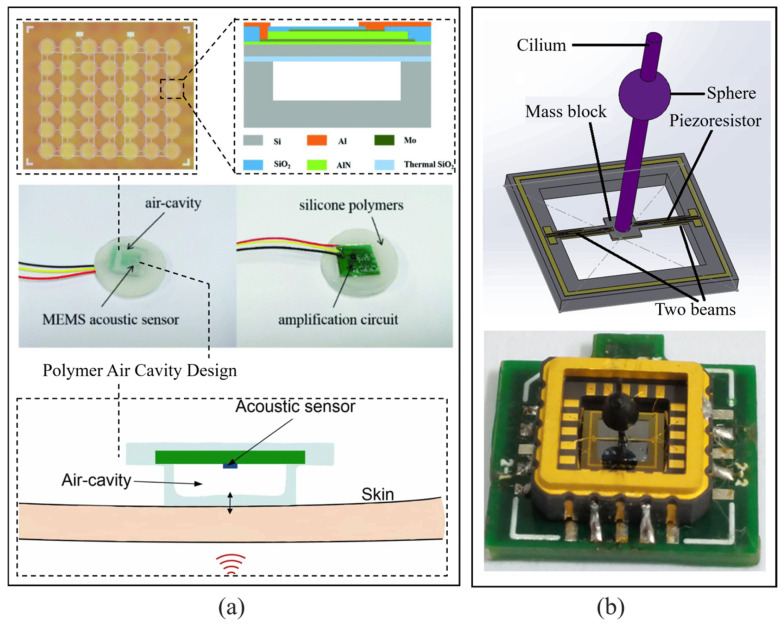
Wearable acoustic sensor based on piezoelectric method. (**a**) Air-silicone composite device for physiological sounds detection [69,72]. (**b**) MEMS bionic hydrophone for heart sound sensing [73].

Piezoelectric MEMS acoustic sensors, with advantages such as integration and low power consumption, are fairly suitable for wearable modes and continuous monitoring. Qu et al. [69] proposed a series of wearable AlN MEMS acoustic sensing devices to monitor heart sounds and detect speech and voice with high accuracy. The devices are packaged by silicone polymers with an air cavity to achieve conformal contact with human skin, and have properties such as light weight, sweatproof capability, resistance to noise, and good stability. Encapsulating rigid and fragile MEMS transducers in flexible materials is a popular solution for human signal detection [70,71]. With the cured silicone, the acoustic sensor along with the circuit board is placed on the top of the cavity and then encapsulated by an additional layer of silicone to produce a monolithic device. Benefiting from the good flexibility of the silicone material, the fabricated device can achieve conformal and direct contact with human skin; therefore, it can be worn like a tattoo, providing a comfortable tool for acquisition of heart sounds. The SNR of their latest device [72] is 13.58 dB, better than the SNR of 6.9 dB from a commercial electronic stethoscope (Figure 6a). This device is small and robust, suitable for wearable physiological sound monitoring. In comparison, the wearable MEMS acoustic sensor proposed by Li et al. [73] is bigger but with better SNR, 15.1 dB, thanks to a biomimetic structure based on a hydrophone and a piezoresistive working method (Figure 6b).

In addition to collecting and calibrating the amplitude of the target acoustic signal, acoustic MEMS sensors can also perform acoustic imaging based on the target’s acoustic characteristics. Rothberg et al. [74] described a low-cost whole-body imaging probe based on silicon-based MEMS ultrasonic sensors directly integrated into complementary metal–oxide–semiconductor-based control and processing electronics, which is the first ultrasound-on-chip platform to be cleared by the Food and Drug Administration for 13 indications. Choi et al. [75] proposed a single-element ultrasound imaging platform, and instead of physically moving the US transducer, the acoustic path is quickly steered by a waterproofed MEMS scanner, achieving real-time imaging. Zhang et al. [76] proposed a cell–element–array design for operation of a PZT MEMS ultrasonic phased-array that can be used to quantitatively characterize the key coupling effects between each pMUT cell, allowing fast 3D volumetric imaging. MEMS technology has greatly facilitated the development of photoacoustic endoscopes and extended the realm of applicability of photoacoustic imaging. As the key component of photoacoustic endoscopes, piezoelectric micromachined ultrasound transducers have been developed and explored for endoscopic photoacoustic imaging applications [77]. Wang et al. [78] presented a dual-frequency piezoelectric micromachined ultrasonic transducer array based on thin ceramic PZT for endoscopic photoacoustic imaging applications. The measured maximum responsivities of the lower- and higher-frequency elements reach 110 nm/V and 30 nm/V at their respective resonances, and the measured cross-couplings of the lower-frequency elements and higher-frequency elements are about 9% and 5%, respectively. MEMS technology has improved the resolution of photoacoustic microscopes and accelerated the development of a handheld photoacoustic microscope. At the same time, an in vivo experiment also proved its potential in biological and clinical applications [79].

## 3. Piezoresistive Method

**Figure 7:**
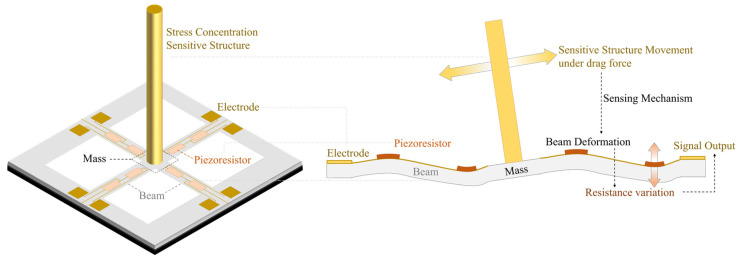
Representative structure and working principle diagram of piezoresistive MEMS hydrophone.

The piezoresistive MEMS acoustic sensor typically features a resonant structure, for example, a cantilever beam structure or a thin film structure, as shown in Figure 7. With the piezoresistive material deposited on the resonant structure, it undergoes transient elastic deformation when receiving pressure waves, leading to a change in the varistor resistance value.

### 3.1. MEMS Acoustic Sensors Based on Piezoresistive Cantilevers

**Figure 8:**
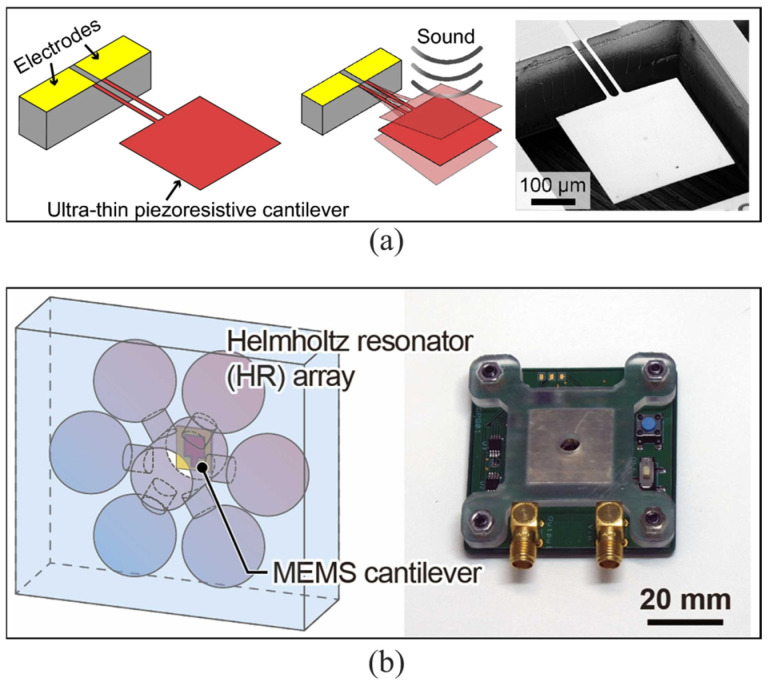
Piezoresistive MEMS acoustic sensor. (**a**) Low-frequency-detectable acoustic sensor using a piezoresistive cantilever [57]. (**b**) Frequency-specific highly sensitive acoustic sensor using a piezoresistive cantilever and parallel Helmholtz resonators [81].

Regardless of the type of device, piezoelectric, piezoresistive, capacitive, or optical, its performance can be improved through optimization of its structure, materials, manufacturing processes, etc. However, devices operating on different working principles possess unique characteristics that inherently confer both advantages and disadvantages. Unfortunately, for frequencies below 20 Hz, the SNR of conventional MEMS-based microphones decreases significantly as the sound frequency decreases [80]. To solve this issue, Kumar et al. [57] developed an acoustic sensor incorporating a piezoresistive cantilever with ultra-high acoustic compliance (Figure 8a). They achieved a resolution of approximately 0.2 mPa, over the frequency range of 0.1–250 Hz, and a sensitivity approximately 40 times higher than that of the previous cantilever device by realizing an ultrathin (340 nm thick) structure with large pads and narrow hinges. The sensitivity of a piezoresistive MEMS acoustic sensor can be improved by using resonant structures, particularly the cantilever beam structure. Wada et al. [81] proposed a frequency-specific highly sensitive acoustic sensor that consists of a MEMS piezoresistive cantilever-type differential pressure sensing element enhanced by a front-mounted parallel Helmholtz resonator array (Figure 8b). Resonant frequencies of the cantilever and Helmholtz resonator are matched to enhance the sensitivity (Δ*R*/*R*/Δ*P* = 2.86 × 10^−3^). Its acoustic pressure resolution is approximately 4 mPa at the resonant frequency (4.5 kHz).

In the field of underwater acoustic sensor technology, the time-varying and spatial characteristics of the ocean sound field make underwater detection very complex. The remote detection of targets is constrained by the high demand for sensor sensitivity, background noise, and low-frequency response ability. The sound field has both a scalar field (sound pressure) and vector field (particle velocity), and both carry sound source information. Therefore, to describe a sound field, both scalar sound pressure and vector particle velocity parameters are required. The acoustic vector sensor is a new type of acoustic sensor that integrates a particle vibration velocity sensor and sound pressure sensor, and can directly measure the vibration velocity of sound particles [82]. They have wide applications in fields such as sound source localization, material acoustic parameter measurement, near-field acoustic holography, and sound intensity measurement [83]. The sensor has the advantages of directional characteristics that are not limited by wavelength, a working frequency range that can cover the audible range, and size comparable to the volume of MEMS microphones [84].

### 3.2. Piezoresistive MEMS Hydrophones with Biomimetic Structure

**Figure 9:**
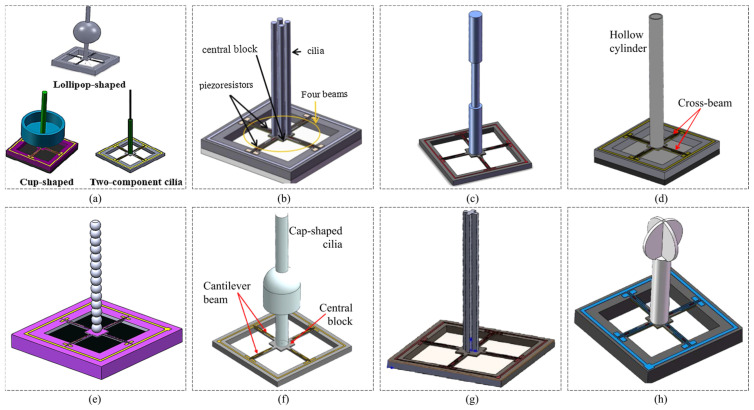
Piezoresistive hydrophones with cilium structure. (**a**) Traditional cilium design in piezoresistive hydrophones [88]. (**b**) CCVH: cilia cluster vector hydrophone [85]. (**c**) DCVH: dumbbell-shaped ciliary vector hydrophone [86]. (**d**) HCVH: hollow cilium cylinder vector hydrophone [87]. (**e**) BCVH: beaded cilia MEMS vector hydrophone [88]. (**f**) CSCVH: cap-shaped ciliary vector hydrophone [89]. (**g**) SCVH: sculpture-shape cilium MEMS vector hydrophone [90]. (**h**) CCCVH: crossed-circle cilium vector hydrophone [91].

When discussing the advancement of piezoresistive MEMS acoustic sensors in recent years, it is essential to highlight the development of biomimetic hydrophones, or underwater acoustic sensors, as shown in Figure 9. These sensors feature stress concentration-sensitive structures inspired by the auditory organs of animals. In the field of MEMS acoustic sensors, significant inspiration has been drawn from biology. Many MEMS acoustic sensors have been designed based on the hearing organs of humans as well as lizards, insects, and fishes. The cilium structure is one of the widely received acoustic signal structural designs in MEMS vector hydrophones for detecting underwater acoustic targets. Devices designed based on this approach are also known as cilium MEMS vector hydrophones (CVHs).

Zhang et al. [85] proposed a cilia cluster MEMS vector hydrophone (CCVH) based on the bionic principle of multiple cilia on fishes’ sense cells. The fabrication of cilia cluster MEMS vector hydrophone is simpler because the cilia have the same properties and integrated process. Compared to a traditional MEMS hydrophone with single cilia design, its sensitivity has been increased by 9.6 dB, reaching up to −183.3 dB at 1600 Hz (0 dB re.1 V/μPa) with the frequency band in the range of 20 Hz–1 kHz. Additionally, the sensitivity is increased by 6 dB per octave. The concave point depth of 8-shaped directivity is beyond 30 dB. Ji et al. [86] developed a dumbbell-shaped ciliary MEMS vector hydrophone (DCVH). Its sensitivity is −186.1 dB (1 kHz, 0 dB = 1 V/μPa), 10.8 dB higher than that of a CVH, and its working frequency band is 20 Hz–1 kHz. The concave point depth exceeds 30 dB. Yang et al. [87] presented a hollow cilium cylinder vector hydrophone (HCVH). The hollow design in the cilium cylinder increases the area that receives sound, so as to improve sensitivity (−185.6 dB (re. 1 V/µPa) at 1250 Hz). Compared to a traditional CVH with a solid cylinder design, the overall sensitivity of the HCVH is improved by 8.7 dB in the 20–1000 Hz range. The hollow design also reduces the hydrophone’s weight to improve its stability and working bandwidth. Lv et al. [88] proposed a beaded cilia MEMS vector hydrophone (BCVH). This beaded cilium structure can enlarge the receiving area for the acoustic wave to improve sensitivity (−183.3 dB at 1 kHz, 0 dB = 1 V/μPa), 13.7 dB higher than that of traditional bionic cilia MEMS vector hydrophones. Meanwhile, it reduced the introduction of additional mass and loss of working frequency band. The depth of the concave point of the BCVH is more than 30 dB, which has a good directivity. Zhu et al. [89] proposed a cap-shaped ciliary vector hydrophone (CSCVH; although it is referred to as CCVH in the original reference article [89], it is referred to as CSCVH here to distinguish it from the cilia cluster MEMS vector hydrophone). The cap-shaped microstructure ensures the working bandwidth of the hydrophone, and increases its sensitivity to −182.7 dB at 1 kHz (1 kHz, 0 dB at 1 V/µPa), which is 14.4 dB better than that of the traditional CVH. The concave point depth of 8-shaped directivity is beyond 30 dB.

Chen et al. [90] designed a sculpture-shape cilium MEMS vector hydrophone (SCVH), whose sensitivity at 1 kHz reaches −184.2 dB (0 dB = 1 V/μPa), 13.1 dB higher than that of a traditional CVH. The working frequency band of the SCVH is 20 Hz–1 kHz, and the depth of the “8”-shaped directivity concave point is larger than 30 dB. Ren et al. [91] proposed a crossed-circle cilium vector hydrophone (CCCVH), which uses two circular structures to increase the receiving area for sound waves and improve sensitivity to −184.5 dB at 1250 Hz (0 dB = 1 V/μPa). By reducing the thickness of the circular structure and adopting a hollow cylinder, the CCCVH also improves its working bandwidth to 20–1250 Hz, with sensitivity reaching −186.7 dB at 1000 Hz, 10.4 dB higher than that of a single-cylinder structure. The depth of the pit exceeds 30 dB at 315 Hz and 630 Hz, indicating that the hydrophone has excellent dipole directivity. Inspired by fish lateral lines, Liu et al. [92] introduced a biomimetic three-dimensional ciliary vector hydrophone (3DCVH). Its detection frequency band is 20–500 Hz. The sensitivities for the three axes are −189 dB at 500 Hz, −189.6 dB at 500 Hz, and −200.9 dB at 500 Hz (0 dB = 1 V/μPa), respectively. The concave point depths are 30.4, 29.8, and 26.9 dB, with the voltage density of self-noise at −106 dB at 500 Hz.

### 3.3. Piezoresistive MEMS Vector Hydrophones with Multiple Biomimetic Cilia Structures

**Figure 10:**
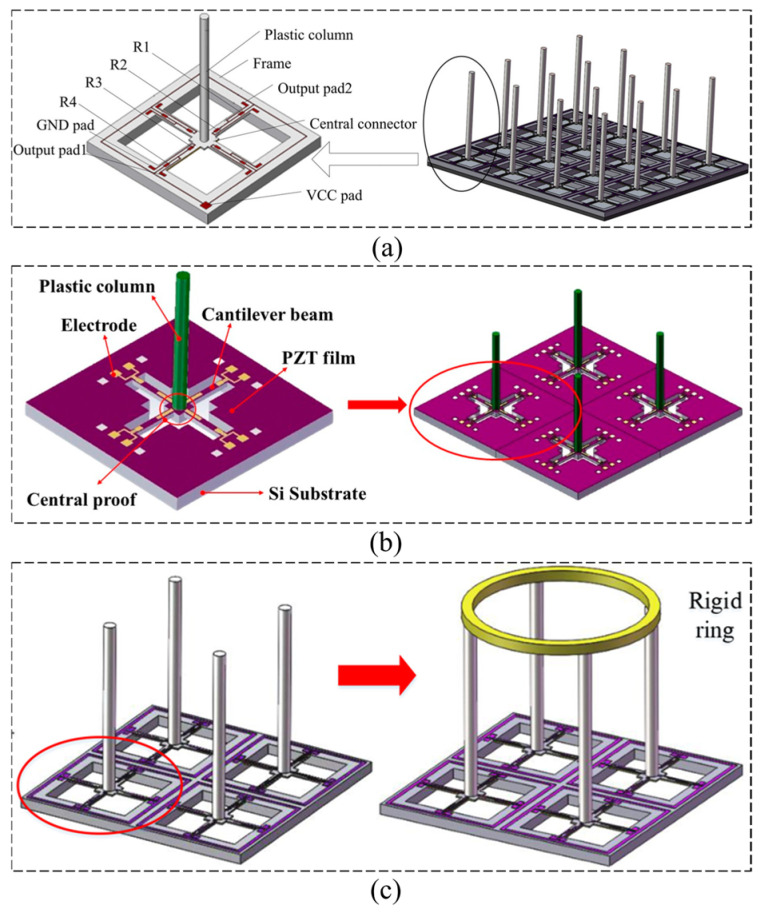
Piezoresistive hydrophones with multiple cilium structure. (**a**,**b**) FUVH: four-unit MEMS vector hydrophone [93,95]. (**c**) FUVH with annulus-shaped structure [94].

In a biomimetic hydrophone, different from the one-unit MEMS vector hydrophone (OPVH), the four-unit MEMS vector hydrophone (FUVH) has multiple cilia or lateral line structures. For example, Zhang et al. [93] proposed a FUVH integrated with multiple sensor units on one chip according to bionics (Figure 10a). The results show that the sensitivity of the four-unit hydrophone is improved by 11.8 dB, and the SNR is improved by 1.9 dB on average. Based on that, Zhang et al. [94] further proposed an FUVH with anulus-shaped ciliary structure (AFUVH). It can realize the complete simulation of the fish lateral line neuromasts structurally and functionally (Figure 10b). Compared to a traditional FUVH, the sensitivity of the AFUVH with annulus-shaped cilia is increased by 5.87 dB, reaching up to −177.53 dB. For another example, Shi et al. [95] developed a FUVH that has a sensitivity of up to −167.93 dB at 1000 Hz (0 dB = 1 V/μPa), 12 dB higher than that of the OPVH. Additionally, the working bandwidth of the FPVH extends through the range of 20 Hz~1200 Hz, exhibiting a good cosine curve with an 8-shape.

The bionic cilium MEMS vector hydrophone has the characteristics of low power consumption, small volume, and good low-frequency response. Nevertheless, there exists the problem of left–right ambiguity in the azimuth estimation of a single hydrophone [96]. To solve this problem, Zhang et al. [96] designed a sound-pressure-gradient hydrophone with two channels. The bionic cilium microstructure is used as the vector channel to collect the sound pressure gradient information, and a piezoelectric ceramic tube as scalar channel to receive the sound pressure information. Its sensitivities can reach up to −188 dB (vector channel) and −204 dB (scalar channel). The problem of left–right ambiguity is solved by combining the sound pressure and sound pressure gradient in different ways. Prabhu et al. [97] presented a biologically inspired MEMS vector acoustic sensor using reduced graphene oxide (rGO) as the thin-film piezoresistive material and Kapton as the substrate. The sensor can detect low-frequency signals spanning from 0.25 Hz to 200 Hz. In air, the sensor exhibits receiving sensitivities ranging from −15.18 dB to −19.75 dB (X-channel) and −13.67 dB to −16.67 dB (Y-channel). In the underwater environment, these values range from −134.40 dB to −138.93 dB (X-channel) and −131.83 dB to −136.93 dB (Y-channel). Furthermore, the device showcases a symmetrical directivity pattern resembling the shape of an “8”, with an approximate sensitivity of −136.66 dB.

Broadly speaking, piezoresistive MEMS acoustic sensors have received a lot of attention in the field of hydrophones, with a focus on the design of biomimetic pressure-sensitive structures. Table 3 gives the performance comparison of recent bionic MEMS vector hydrophones based on ciliary structures. Although there is no fundamental difference between cilia structure and fish lateral line structure, it gives piezoresistive MEMS acoustic sensors more space and freedom in geometric design, especially after introducing FUVH composed of multiple sensitive units. These devices have achieved good results in sensitivity, working frequency bandwidth, and directionality. Comparatively speaking, although different biomimetic designs were used, they did not show decisive differences in performance parameters such as sensitivity. However, with the introduction of more innovative structures, piezoresistive MEMS hydrophones are expected to achieve higher performance and practicality.

## 4. Capacitive Method

**Figure 11:**
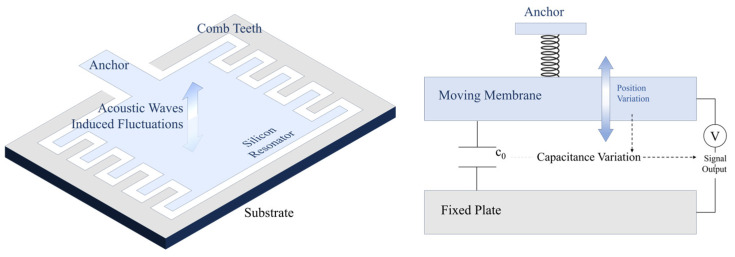
Representative structure and working principle diagram of capacitive MEMS acoustic sensors.

Unlike piezoelectric or piezoresistive sensors, capacitive MEMS acoustic sensors do not require the resonant structures on functional materials, as shown in Figure 11, allowing for more freedom in constructing complex two-dimensional geometries and adjusting capacitance changes under acoustic input.

### 4.1. Capacitive MEMS Microphones

MEMS microphones represent one of the most prevalent and well-developed applications within MEMS technology, with capacitive MEMS microphones exemplifying this category. The capacitive MEMS microphones are generally composed of MEMS micro capacitive sensors, micro integrated conversion circuits, acoustic cavities, and RF anti-interference circuits. The MEMS micro capacitor electrode head includes a silicon diaphragm that receives sound and a silicon back electrode (Figure 11). The silicon diaphragm can directly receive audio signals, which are transmitted to the micro integrated circuit through the MEMS micro capacitor sensor. The micro integrated circuit converts and amplifies high-impedance audio electrical signals into low-impedance electrical signals, which are then filtered by an RF anti-noise circuit to output electrical signals that match the pre circuit, completing the acoustic electrical conversion. By reading electrical signals, sound recognition can be achieved.

Prior to the advent of microelectromechanical systems (MEMS) acoustic sensors, conventional electret microphones were widely utilized in consumer electronics, including smartphones, headphones, and computers. The popularity of smartphones has led to the widespread application of MEMS acoustic sensors. As the manufacturing processes for MEMS acoustic sensors have matured and shipment volumes have steadily increased, the resulting decline in terminal costs has facilitated the widespread adoption of MEMS acoustic sensor applications, extending their use from high-end to mid- and low-end smartphones. The use of external headphones and tablets is also increasing, and they are further applied in fields such as medical electronics, automotive electronics, wearable devices, and the Internet of Things.

Ozdogan et al. [98] developed a capacitive MEMS microphone with a levitation-based electrode configuration. This electrode scheme enables capacitive MEMS sensors that could work for large bias voltages without pull-in failure. They managed to create robust sensors that work properly at high DC voltages, which is not feasible for most of the conventional parallel-plate electrode-based microscale devices. The sensitivity of this initial design was measured to be 16.1 mV/Pa at 200 V bias voltage, and the bandwidth was from 100 Hz to 4.9 kHz. Sant et al. [99] proposed a new MEMS microphone based on a sealed-dual membrane (SDM) design paired with the latest generation digital read-out ASIC. The SDM design reduces significantly the magnitude of one of the main noise contributors by moving the air gaps to a sealed low-pressure chamber. State-of-the-art noise performance is achieved thanks to significant optimizations both on the MEMS as well as on the ASIC side. It achieves an SNR of 72 dB(A) supporting an acoustical overload point (AOP) of 130 dB SPL. Yin et al. [100] presented a MEMS resonator with capacitive transduction as an acoustic sensor, intended for cantilever-enhanced photoacoustic spectroscopy. Its silicon resonator has dense comb teeth for area-variable capacitive detection. The anchor height is increased to 260 μm to reduce gas damping effects on the resonator motion and successfully resolves the capacitance detection sensitivity and motion damping trade-off. Experimental results exhibit a maximum sensitivity of 3749 mV/Pa at the resonant frequency of 1870 Hz with a 15 V bias voltage. The equivalent noise has a peak value of 7.9 μPa/√Hz.

**Figure 12:**
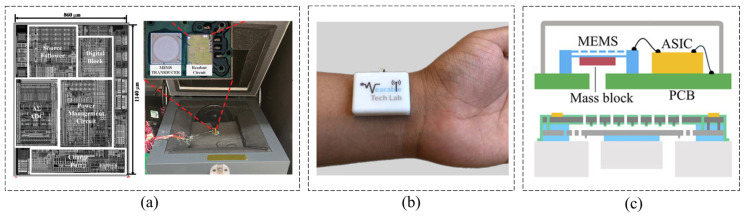
Capacitive MEMS Microphone. (**a**) Low-power digital capacitive MEMS microphone based on a triple-sampling delta-sigma ADC with embedded gain [101]. (**b**) Wearable capacitive MEMS microphone for cardiac monitoring at the wrist [102]. (**c**) Capacitive MEMS stethoscope with anti-stiction-dimple array design in the diaphragm and the backplate for highly reliable heart or lung sounds detection [105].

In addition to the device design itself, the optimization of sampling technology can also suppress noise, improve resolution, and enhance the maturity of the entire sensing system. Lee et al. [101] presented a triple-sampling technique, resulting in a readout circuit with noise performance comparable to recent designs, but with a reduced power requirement and increased sensitivity (Figure 12a). A triple-sampling ΔΣ ADC can replace the programmable gain amplifier commonly used in the readout circuit for a digital capacitive MEMS microphone. The input voltage can then be multiplied by subtracting a further half-period delayed differential input and using the feedback capacitor of the DAC as a sampling capacitor. A MEMS microphone incorporating this readout circuit, fabricated in a 0.18 μm CMOS process, had an area of 0.98 mm^2^ and achieved an A-weighted SNR of 62.1 dBA at 94 dBSPL with 520 μA current consumption, to which triple-sampling was shown to contribute 4.5 dBA.

With the maturation of and cost reduction in capacitive MEMS microphone technology, it is increasingly supplanting traditional microphones and similar acoustic devices in the market, thereby expanding its range of applications. Traditionally, auscultation involves using the ears or a stethoscope to detect heart or respiratory sounds, enabling medical professionals to diagnose the presence or absence of lesions in related organs by analyzing the characteristics and changes in these sounds. Currently, the detection of heart sound signals still relies on doctors using traditional stethoscopes, which depends heavily on the doctors’ clinical experience and is inherently subjective. However, advancements in electronic technology, biomedical engineering, and signal processing and analysis have led to the widespread adoption of electronic stethoscopes in modern auscultation. These devices amplify faint heart or lung sounds into electrical signals (Figure 12b), thereby enhancing the listening experience and improving diagnostic precision [102]. For an electronic stethoscope using a traditional coil microphone, the air gap between the microphone diaphragm and the stethoscope diaphragm allows the microphone to absorb excessive environmental noise, resulting in ineffective sound energy transmission [103]. MEMS acoustic sensors can adjust the vibration of sensitive units under different frequencies of sound wave excitation through microstructure design, achieving good sound energy transmission efficiency and reducing distortion in micro scale spaces such as stethoscopes [104].

Zheng et al. [105] designed a novel acoustic-vibration capacitive MEMS microphone as an electronic stethoscope for the collection of the characteristics and frequency spectrum of low-frequency cardiac vibration signals (Figure 12c). In this microphone, the structure of the anti-stiction-dimple array is designed and deployed at the bottom of the diaphragm and the backplate to avoid the risk of sensor failure by vibration stiction. Typical characteristic results show that the open-circuit sensitivity of the microphone is 12.63 mV/Pa (37.97 dBV/Pa) at 1 kHz (with 94 dB as the reference sound level). The total harmonic distortion and acoustic overload point are 0.21% and 121.2 dB sound pressure level, respectively. As Zheng et al. showed in their work [105], the optimization of the MEMS microphone backplate can significantly affect its performance. For example, Shubham et al. [106] proposed a backplate design with center and peripheral protrusion structure, which can increase the effective area, linearity, and sensitivity. A center and eight peripheral protrusions extend from the backplate, leading to a 48% increase in the effective area with respect to a simply supported diaphragm without the center protrusion. The device also has a semi-constrained polysilicon diaphragm with flexible springs that are simply supported under bias voltage. The flexible springs attached to the diaphragm reduce the residual film stress effect more effectively compared to constrained diaphragms. With an applied bias, the microphone has a sensitivity of −38 dB (re. 1 V/Pa at 1 kHz) and an SNR of 67 dBA measured in a 3.25 mm × 1.9 mm × 0.9 mm package including an analog ASIC.

### 4.2. Recent Progress in Theoretical Models for Capacitive MEMS Acoustic Sensors

To effectively analyze and predict how the structure or mechanical properties of the backplate influence device performance, precise simulation models are necessary. Peng et al. [107] presented a lumped-parameter model (LPM) providing a deeper understanding of the compliant backplate in capacitive MEMS microphones. They not only modeled the backplate as vibrating but also considered the coupling effect between the mechanical and electrical domains. The theoretical derivations using Lagrange equations show how backplate motion can impact the microphone’s performance, and the model with electrical coupling of the vibrating backplate effectively captures the sensitivity dip resulting from the backplate resonance. A backplate that is overly compliant can narrow the operating frequency range and increase the likelihood of experiencing pull-in. As with the backplane theoretical model proposed by Peng et al. [107], a dedicated analysis model, which is crucial for the design of capacitive MEMS microphones, can accurately simulate the mechanical behavior and electrical output of devices under pressure waves, and thus accurately predict parameters such as microphone sensitivity and signal-to-noise ratio. For example, Shubham et al. [108] proposed a behavioral nonlinear system-level modeling approach for MEMS capacitive microphones. The models capture diaphragm displacement and capacitance change over large pressure ranges for a simply supported circular plate. This can be coupled with the electrostatic force, due to the applied bias voltage, and converted back to pressure, thereby realizing a feedback loop in the circuit model. The nonlinear model capability is extended to predict capacitance-bias behavior and estimate pull-in of the MEMS device. The microphone sensitivity, signal-to-noise ratio, and harmonic distortions are accurately predicted using this nonlinear large signal model. For another example, Anzinger et al. [109] provides an analytical non-linear model for the capacitive transduction in MEMS transducers with perforated counter-electrodes, especially applicable to capacitive MEMS microphones. Starting from an electrostatic description of a perforated unit cell of the transducer, analytical formulations of the variable capacitance and electrostatic forces are derived, accounting for the deflection profile of a clamped circular plate. A lumped implementation into conventional circuit simulation tools is enabled via behavioral modeling based on hardware description languages, such as Verilog-A. The resulting model finally enables both a small- and large-signal analysis of capacitive MEMS microphones, precisely accounting for non-linearities in the capacitive transduction. This allows one to simulate the harmonic distortion of the microphone’s output signal and account for electrostatic spring-softening in simulations of its bias voltage-dependent sensitivity.

### 4.3. Capacitive MEMS Microphones with Biomimetic Design

**Figure 13:**
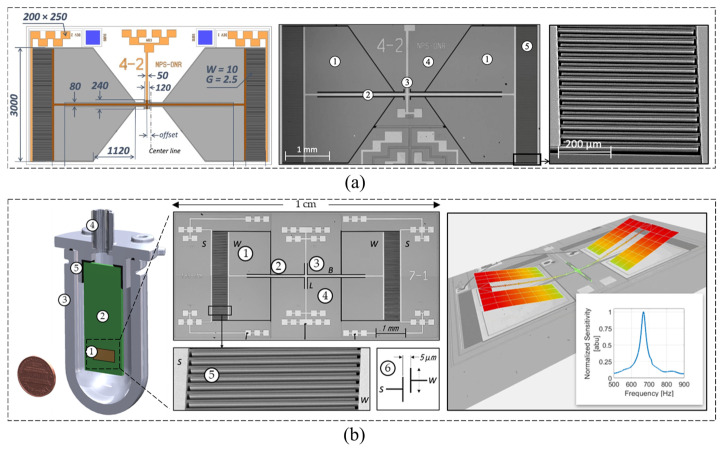
Capacitive MEMS microphone with biomimetic design. (**a**) Dual-band MEMS directional acoustic sensor for near-resonance operation [110]. (**b**) Directional-resonant MEMS acoustic sensor and associated acoustic vector sensor [111]. Both (**a**,**b**) are inspired by the tympana configuration of the parasitic fly *Ormia ochracea*. The circled numbers in (**b**) are used to distinguish different structures.

The intricate structure of animal organs can provide numerous inspirations for the design of MEMS acoustic sensors. In piezoresistive devices, it can be a biomimetic pressure wave receiver based on cilia or fish lateral lines. In capacitive devices that emphasize the spatial variation between conductive planes, more inspiration comes from animal eardrums. Alves et al. [110] reported a MEMS directional sensor inspired by the tympana configuration of the parasitic fly *Ormia ochracea* (Figure 13a). By means of breaking the symmetry of a pair of coupled membranes, two independent bending vibrational modes can be excited. The sensor exhibits, at resonance, mechanical sensitivity around 6 μm/Pa and electrical sensitivity around 13 V/Pa. The computed average signal-to-noise ratio in the pass band is about 91 dB. Ivancic et al. [111] developed an *Ormia ochracea* tympana-inspired MEMS acoustic vector sensor (AVS) for the detection and location of quiet or distant acoustic sources of interest (e.g., gunshots and drones). The sensor demonstrated a maximum SNR of 88 dB with an associated sensitivity of −84.6 dB re 1 V/μPa (59 V/Pa) (Figure 13b). The AVS demonstrated an unambiguous, 360-degree, in-plane, azimuthal coverage and was able to provide an acoustic direction of arrival to an average error of within 3.5 during field experiments. Table 4 lists the performances of several recent capacitive MEMS acoustic sensors with different features.

Piezoelectric MEMS acoustic sensors have gradually developed and have been widely commercialized, expanding their functionality. Yang et al. [112] presented a digital capacitive MEMS microphone for speech recognition with a fast wake-up feature. The proposed deglitching technique is applied to prevent the sound activity detector from responding to non-acoustic signals such as glitch signals. An auxiliary low-dropout regulator is used to further reduce the wake-up time. This microphone achieves an A-weighted SNR of 62.8 dBA and an acoustic overload point of 119.4 dB in sound pressure level.

Compared with other methods, MEMS acoustic sensor capacitive methods are more advanced and widely used in fields such as microphones. Unlike piezoelectric or piezoresistive devices, capacitive devices exhibit higher flexibility in structural design, due to the absence of limitations in piezoelectric materials. They can further adjust performance through micro mechanisms such as cantilever beams, backplates, micro springs, and micro levers. The advancement of this technology is attributable not only to the improved device structure, performance, and reliability, but also to the development of theoretical analysis methods, which have been significantly bolstered by advancements in simulation models.

## 5. Optical Method

**Figure 14:**
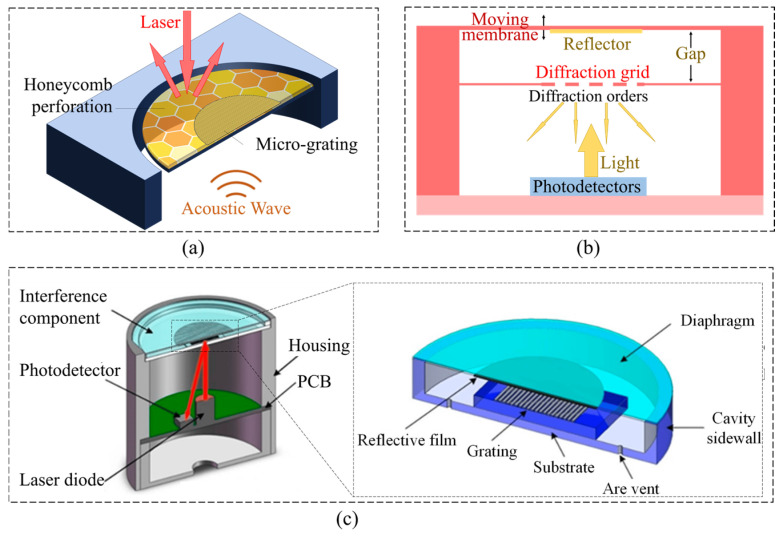
MEMS acoustic sensor based on optical grating interferometer. (**a**) A grating interferometer design by a diffraction grating integrated backplate and a pressure-sensitive diaphragm [117]. (**b**) Design of a MEMS optical microphone transducer based on light phase modulation [120]. (**c**) Grating interferometer design with short-cavity structure and grating-on-convex-platform structure [118,119].

While MEMS technology is progressively advancing and becoming widely commercialized, the scaling laws set a limit on MEMS acoustic sensors’ sensitivity and sensing resolution, especially for the devices using electronic readout techniques [113], such as capacitive [114], piezoresistive [115], and piezoelectric [116] methods, hindering their use in high-resolution applications such as tactical and strategic-grade navigation, underwater acoustics, and microbiology, forcing the device to adopt special processes or structures to suppress noise [58]. Introducing optical components into integrated MEMS devices and applying optical readout technology can effectively solve this problem. In this section, we primarily examine the recent developments in three types of optical MEMS acoustic sensors, including the ones based on a grating interferometer (Figure 14a,b), Fabry–Perot interferometer, and micro-opto-mechanical structure.

### 5.1. Optical MEMS Acoustic Sensors Based on Grating Interferometer

A capacitive MEMS microphone in a small package encounters performance limits due to the gas damping effects on the resonator motion, which lead to a trade-off between the capacitance detection sensitivity and motion damping [100]. To address this issue, Li et al. [117] developed an SOI-MEMS acoustic sensor based on an optical grating interferometer. The designed sensor chip forms a grating interferometer by a diffraction grating-integrated backplate and a pressure-sensitive diaphragm (Figure 14a). The sensor device was fabricated on a silicon-on-insulator (SOI) wafer using complementary metal–oxide–semiconductor (CMOS) compatible processes. The frequency response is relatively consistent with that of a commercial reference microphone for the audible range. The SNR is about 43 dB at a frequency of 1 kHz. Zhang et al. [118] reported a MEMS acoustic sensor based on a grating interferometer. They utilized a short-cavity structure to reduce the impact of temperature on the cavity length in order to improve its stability against environmental temperature variations. Additionally, through holes were designed in the substrate of the grating to reduce the air damping of the short-cavity structure. The sensitivity of the acoustic sensor is up to −15.14 dB (re. 1 V/Pa at 1 kHz). The output signal of the high-stability acoustic sensor was almost unchanged as the environmental temperature ranged from 5 °C to 55 °C. Based on this sensor, Zhang et al. [119] further developed an acoustic sensor with a glass-based grating interference component. The grating-on-convex-platform structure of the glass-based components were designed and developed (Figure 14c). As a result, they obtained a sensitivity of 0.776 V/Pa at 1 kHz, and the spectrum of its sensitivity was flat from 50 Hz to 8 Hz, with a deviation of less than 1.5 dB and a sensitivity of 0.408 V/Pa at 20 Hz.

Acoustic sensing through optical transduction represents a promising alternative to the conventional capacitive sensing used in MEMS microphones, especially when aiming at ultralow-noise applications. In fact, the traditional acoustic to electrical transduction stages are decoupled by the intermediate conversion of the signal into the optical domain. As a result, the mechanical design of the sensor has no direct influence on the electrical readout performance [120]. This allows for a significant reduction in the MEMS transducer noise through aggressive acoustically semi-transparent stator designs that represent one of the limits of the standard capacitive technologies (Figure 14b). Milleri et al. [120] reported a design and the modeling of the sensing elements of a MEMS optical microphone. In their work, the main second-order effect observed was studied, isolated, and reproduced both in measurements and simulations. A fundamental design improvement was deduced, i.e., the reduction in the light reflections in the structure (especially in the Bosch cavity of the MEMS), e.g., with anti-reflective coating of the chips. A reliable and powerful modeling platform was developed that allows for prediction of the performance of the system also in the case of high-volume production with the unavoidable fabrication tolerances.

### 5.2. Optical MEMS Acoustic Sensors Based on Fabry–Perot Method

**Figure 15:**
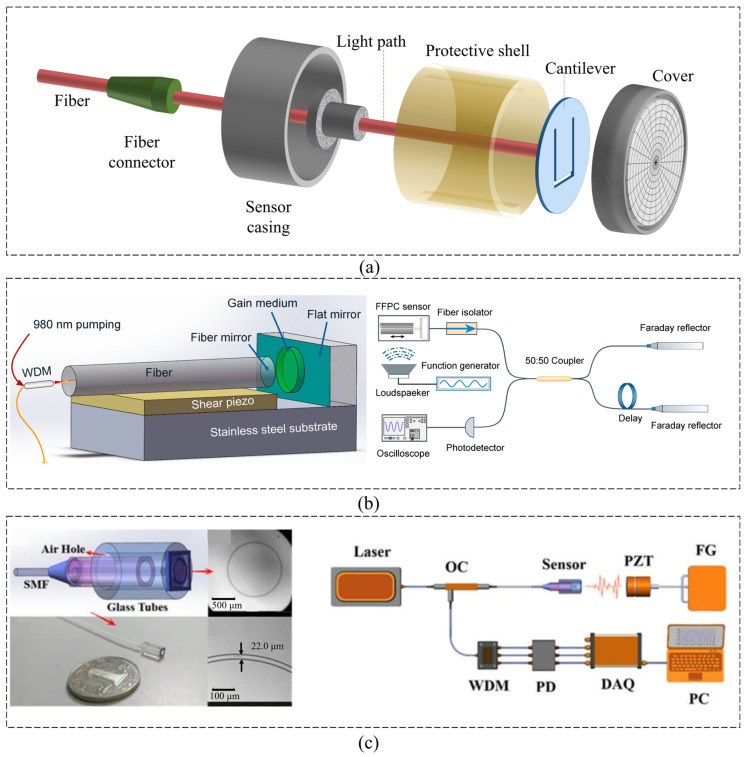
MEMS acoustic sensor based on Fabry–Perot method. (**a**) A typical structure of Fabry–Perot MEMS acoustic sensors. (**b**) An acoustic sensor based on active fiber Fabry–Pérot microcavities [21]. (**c**) An application in the detection and position of partial discharge [122].

The successful assembly of optical interferometric microsensors on a large scale hinges on the precise and repeatable alignment of their small interferometers (Figure 15a). Behrad Habib Afshar et al. [113] reported a fiber-optical acoustic sensor (FOAS) that utilizes a self-aligning two-wave interferometer and exhibits a record pressure resolution. The sensor consists of a circular, spring-loaded diaphragm that vibrates in and out of the plane of a stationary substrate when exposed to acoustic pressure. This piston-like differential motion between the diaphragm and the adjacent substrate is detected interferometrically with a free-space laser beam, delivered by a single-mode fiber, that straddles the diaphragm–substrate boundary. The sensor exhibits a self-noise limited by residual excess noise below 300 Hz and by the very small thermomechanical noise of the diaphragm above 300 Hz. Its average pressure resolution of 215 nPa/√Hz between 40 Hz and 4 kHz establishes a new record. Gong et al. [121] presented a silicon cantilever-based FOAS. The length, width, and thickness of the rectangular cantilever are 530 μm, 200 μm, and 3 μm, respectively, and its resonant frequency is 14,820 Hz with a sensitivity of 950 nm/Pa. An ultra-high-speed absolute cavity length demodulation method was adopted using a complementary metal oxide semiconductor (CMOS) spectrometer and an 850 nm superluminescent light emitting diode (SLED). A modified Buneman frequency estimation and total phase demodulation algorithm was adopted. The frequency response curve showed a relatively flat trend from 20 Hz to 13 kHz. The sensor exhibited good linearities at different frequencies while the applied acoustic pressure was increased from 0 Pa to 2.5 Pa. Experimental results indicate that the minimum detectable pressure (MDP) of the proposed FOAS is calculated to be 25.68 μPa/√Hz at the frequency of 13 kHz.

As a comparison, the Fabry–Perot silicon cantilever-based FOAS proposed by Guo et al. [20] has a lower pressure sensitivity but better MDP. To achieve ultrahigh-sensitivity acoustic detection, a white light interference (WLI) demodulation system based on an amplified spontaneous emission (ASE) source was used to demodulate the cavity length of the sensor. The acoustic pressure sensitivity of the sensor was measured to be 1.753 μm/Pa at a frequency of 1 kHz and 28.75 μm/Pa at the resonance frequency of the cantilever. Experimental results indicated that the MDP level of the fabricated sensor was 0.21 μPa/√Hz at 1 kHz. The silicon-based FOAS proposed in this article demonstrated its ability to detect ultraweak acoustic signals due to its extremely high sensitivity. Xin-Gao et al. [21] demonstrated an active acoustic sensor based on a high-finesse fiber Fabry–Perot micro-cavity with a gain medium (Figure 15b). The sensor is a compacted device lasing around 1535 nm by external optical pumping. The acoustic pressure acting on the sensor disturbs the emitted laser frequency, which is subsequently transformed to beat signals through a delay-arm interferometer and directly detected by a photo-detector. In this configuration, the sensing device exhibits a high sensitivity of 2.6 V/Pa and a noise equivalent acoustic signal level of 230 μPa/√Hz at a frequency of 4 kHz. Experimental results provide a wide frequency response from 100 Hz to 18 kHz. As the sensor works at communication wavelengths and the output laser can be electrically tuned in the 10 nm range, a multi-sensor network can be easily constructed with dense wavelength division multiplexing devices.

In addition to improving performance, researchers have also focused on the application of Fabry–Perot FOAS. Liu et al. [122] designed an optical fiber-based ultrasonic sensor and applied it to the detection and position of partial discharge (PD) (Figure 15c). In the PD detection experiment, the MDP of the FOAS achieved 0.455 μPa/√Hz. In addition, four ultrasonic sensors were arranged around the ultrasonic source to conduct localization experiments. The results show that the system has excellent localization performance in the x, y, and z directions.

### 5.3. Optical MEMS Acoustic Sensors Based on Micro-Opto-Mechanics

In addition to the grating interferometer and Fabry–Perot methods, researchers have also proposed multiple micro-opto-mechanical acoustic sensors in recent years. Xiong et al. [123] reported a compact yet highly sensitive all-optical acoustic pressure sensor. It consists of a micro-opto-mechanical silicon cantilever beam, with a length of 9.5 mm, a width of 2.5 mm, and a thickness of 10 μm, and integrated with a rib waveguide located on the top of the cantilever beam. Two grooves are created on the same substrate and aligned in line with the rib waveguide. Two optical fibers are then fixed into the two pre-aligned grooves on both sides of the rib waveguide, separately, for optical signal coupling in and out. The results show an acoustic pressure detection sensitivity of 8.34 V/Pa with a minimum detectable acoustic pressure of 35 nPa/√Hz at 150 Hz. Lorenzo et al. [124] proposed a compact hydrophone capable of measuring acoustic signals from cardiomyocytes. This hydrophone consists of a nanofabricated photonic-crystal diaphragm externally mounted to the facet of an optical fiber to form a pressure-sensitive Fabry–Pérot cavity. The hydrophone can operate in small liquid volumes less than 5 mm deep and incorporates a microchannel to vent air during immersion. The venting channel is designed to optimize bandwidth and sensitivity. Modeling and experimental results in water show a bandwidth from 50 Hz to 18 kHz and a minimum detectable pressure of 3 μPa/√Hz. The hydrophone also demonstrated sensitivity to simulated bio-acoustic sources with nanometer-scale displacements. Table 5 summarizes the performances of recent optical MEMS acoustic sensors based on different methods.

Overall, in optical MEMS acoustic sensors, interferometer-based readout methods are still mainstream and widely used in structures including Fabry–Perot cavities. An optical fiber readout method allows the device to have a rather simpler micromachine structure, without comb teeth or air chamber, and less gas damping effects on the resonator motion. Instead, it requires gratings and the bonding between glass and silicon, after considering the transparency and reflectivity among layers. Compared to the others, optical MEMS acoustic sensors can have a very low noise floor, which allows them to identify weaker acoustic signals and have better pressure resolution.

## 6. Development Trends

Even before the introduction of MEMS technology, acoustic sensors were widely used for detecting sound pressure, mass, acceleration, and properties of solid or fluid samples, as well as for sensing biological or medical information in fields such as disease diagnosis [125]. With the advancement of microfabrication and microscale analysis technology, MEMS acoustic sensors have been widely applied in various fields. The most typical applications of MEMS acoustic sensors are in microphones [98,99,100,101,102,103,104,105,106] and hydrophones [85,86,87,88,89,90,91,92,93,94,95,96,97], utilizing their advantages in sensitivity or noise resolution to replace traditional acoustic sensors. Although the MEMS acoustic sensors based on various sensing mechanics can all achieve high-precision acquisition of acoustic energy, different device types have developed unique applicability for specific fields.

### 6.1. MEMS Acoustic Sensor Applications and Trends

As shown earlier, capacitive MEMS acoustic sensors dominate in the microphone field and occupy a large market share, while interest in piezoresistive MEMS acoustic sensors is largely focused on the hydrophone, and piezoelectric methods have been widely applied in the biomedical field. The differences in terms of application stem from the characteristics of different sensing principles. The capacitive principle has the characteristics of high sensitivity, high signal-to-noise ratio, and low noise, which enables it to achieve high-performance sensitive units [126]. Considering that devices based on some other sensing method can also achieve high sensitivity and wide dynamic ranges, the performance advantage is not the biggest reason for the widespread popularity of capacitive MEMS microphones. Compared to other methods, capacitive devices do not require piezoelectric or resistive materials, nor do they require additional optical paths [98], which allows them to be constructed through simpler processes and at lower costs, thus gaining higher competitiveness in popular consumer electronics such as microphones. In addition, without the limitations of functional materials and optical paths on the process or structure, capacitive MEMS devices can adjust their performance indicators through mechanical means such as micro levers and micro springs, giving them higher degrees of freedom in geometric design.

In fact, microphones, as an important application area for acoustic transducers, are the focus of interest for many MEMS acoustic sensors. Piezoelectric devices have the advantage of high reliability and are also considered for microphones as an important application area. However, they require the construction of piezoelectric thin films through complex masking and etching to enhance sensitivity [30,31,32,33,34,35,36,37,38,39], and sometimes use microchannels to improve the signal-to-noise ratio [51], which limits their advantages in terms of cost and structural simplicity, and weakens their leverage as consumer electronics. However, the negative piezoelectric effect of piezoelectric materials enables their acoustic sensors to also be used as speakers, and their positive piezoelectric effect gives them the advantage of being wireless and passive. This makes them highly suitable for low-power hydrophones used for underwater detection, or wireless acoustic sensors for wearable or implantable microsystems [69,70,71,72,73], including microphones, stethoscopes, hearing aids, and artificial organs. In recent years, the MEMS piezoelectric microphone has been commercialized and is gradually taking up market share in consumer electronics due to its exceptionally low power consumption, which allows constant standby [126]. In the future, piezoelectric MEMS acoustic devices, with their wireless and passive characteristics, as well as the ability to integrate sensing, communication, and actuation on one sensitive structure, will see greater development in fields such as implantable devices and bioMEMS.

Optical MEMS acoustic sensors, with their advantages of high sensitivity, wide dynamic range, and immunity to electromagnetic interference, should have had the widest and deepest applications among numerous devices. External light sources, interferometers, microcavities, and optomechanical structures [117,118,119,120,121,122,123,124] pose difficulties in their manufacturing, packaging, and integration, limiting their target positioning. Among them, fiber-based optical systems have high universality as high-precision readout tools and are widely used for micro displacement detection in various scenarios. The introduction of optical paths eliminates the need for physical connections such as electrodes or functional materials for signal acquisition, making optical MEMS acoustic sensors uniquely applicable in principle. Based on these characteristics, optical MEMS acoustic sensors are more suitable for high-precision detection in extreme environments, and considering system costs, they have greater competitiveness in industrial rather than consumer electronics. As a comparison, piezoresistive MEMS acoustic sensors, especially devices based on four cantilever beams and piezoresistive plates, can rely on universal sensing principles and exchangeable pressure-sensitive cilia structures to acquire much more freedom in structural design [85,86,87,88,89,90,91], leading to extensive research in the field of hydrophones.

The scale effect and integrated structure allow MEMS acoustic sensors to have optimized performance parameters, with the most representative being optical acoustic sensors that integrate thin-film structures, interferometers, and micro optical paths in one device and achieve precise acoustic pressure detection with optical readout methods [117,118,119,120]. By miniaturizing interferometers or Fabry–Perot cavities to the microscale, these devices, compared to traditional acoustic sensors based on optical readout methods, have significant size advantages, allowing them to be applied to smaller systems [127,128]. However, the size advantage brought by the scale effect is also relative. Optical MEMS acoustic sensors have a looser structure compared to piezoresistive or piezoelectric acoustic sensors, which is a concession for constructing optical paths [121,122]. Most notably, capacitive MEMS acoustic sensors with higher integration and technological maturity can be integrated into smaller systems, such as microphones in smart electronic products [129,130]. Just as Pip Knight summarized, intelligent instruments like smart speakers that can register our everyday commands have become commonplace in homes around the world, while the acoustic sensors inside these devices are far flung from the first microphone, and the technology is still evolving [131].

### 6.2. Medical Applications

**Figure 16:**
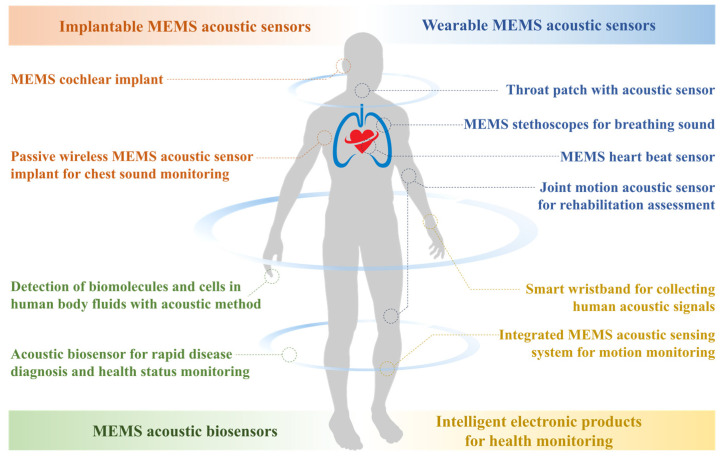
Applications of MEMS acoustic sensors in biomedical field.

In addition to the aforementioned applications, MEMS acoustic sensors are also widely used in fields such as human health and biomedicine, as shown in Figure 16. The increasing attention to health issues [132] and rising demand in the market have expanded applications for MEMS acoustic sensors, and the advancement of biomedical technology and supporting industries has also promoted the application of MEMS acoustic sensors in interdisciplinary fields. Traditional acoustic sensors are also used for biomedical applications such as disease diagnosis, but in limited fields, such as B-mode ultrasonography [133] or stethoscopes [105], as derivatives of ultrasound imaging [134] or microphone devices [135]. Indeed, MEMS acoustic sensors can replace traditional medical acoustic sensors such as stethoscopes [69,70,71,72] and provide advanced acoustic methods for precise disease detection or biochemical signal sensing. But more importantly, as emphasized in this section, the scale effect and the accompanying size advantages will effectively help MEMS acoustic sensors to be integrated into smaller systems with lower power consumption, which is also the case in the biomedical field. This enables MEMS acoustic sensors to be applied in scenarios that are beyond the reach of traditional devices.

Wearable intelligent systems, as a direct, safe, and non-invasive means of health monitoring, have become a breeding ground for the development of various MEMS sensors [136]. Among them, MEMS acoustic sensors play an important role as portable stethoscopes to achieve long-term monitoring of signals such as breathing sounds, pulse, and heartbeat [72], for applications like physical condition monitoring, motion recognition, disease prediction, and health warning [137]. Compared to traditional devices, MEMS acoustic sensors can achieve more functions in wearable applications. For example, a multimodal sensing system of contact and airborne measurement of joint acoustic emission based on MEMS acoustic sensors can realize joint rehabilitation assessment following musculoskeletal injury [138].

Another important manifestation of the small size and low power consumption advantages of MEMS acoustic sensors is in implantable devices. For example, the cochlear implant based on MEMS acoustic sensors [139] can achieve precise sound collection with an integrated and totally implantable structure and help patients to regain hearing and overcome discomfort, inconvenience, and social stigma [140]. The completely passive, wireless MEMS acoustic sensor, packaged with RF communication devices such as surface acoustic wave interdigital transducers, breaks new ground with further improved integration and reduced power consumption requirements. It can be used for monitoring breathing sounds in apnea patients, monitoring chest sounds after cardiac surgery, feedback sensing in compression vests used for respiratory ventilation, and monitoring chest sounds in neonatal care [141]. The integrated structure and passive sensing mechanics of surface acoustic wave transducers give these sensors unique advantages as small passive wireless microphones [142]. In addition to serving as a wireless communication device, MEMS surface acoustic wave transducers can also be used for biosensors. The resonance mode of surface acoustic wave devices makes them highly sensitive to frequency shift and capable of producing high-precision outputs under the mass effect of small loads, making them suitable for detecting micro-nano biomedical targets such as antibodies [143] and bacteria [144]. Although different from other forms of MEMS acoustic sensors, MEMS surface acoustic wave sensors are not only widely used in integrated MEMS acoustic systems, but can also serve as a high-performance passive wireless device, or as a supplemental method providing detection means for molecular-level samples [145]. Sezen et al. [141] introduced a wireless, battery-less surface acoustic wave MEMS microphone with a pulse-modulated surface acoustic wave-based sensing strategy, designed for monitoring breathing sounds in apnea patients, monitoring chest sounds after cardiac surgery, and feedback sensing in compression vests used for respiratory ventilation. Compared with other piezoelectric MEMS acoustic sensing devices, acoustic MEMS biosensors place more emphasis on biocompatibility, surface compatibility with biomaterials, and noise resolution within fluids [146]. By constructing a Lab on a Chip device, acoustic MEMS biosensors can detect biological potentials and chemical drugs at the microscale through the frequency variation caused by principles like the mass effect [147]. Nowadays, MEMS surface acoustic wave biosensors are applied to detect various diseases, pathogens, and other biomolecules, such as HIV [148], cancer cells [149], *E. coli* [150], *Pseudomonas aeruginosa* [151], penicillin [152], and growth factor [153,154].

Overall, the applied research on MEMS acoustic sensors in health monitoring mainly focuses on collecting acoustic signals from vocal human organs [71], visceral organs [137], and limb movements [138], or on the use of implantable sound-sensitive devices to replace auditory organs [139]. In these applications, flexible materials, biocompatible materials, and bioinformatics algorithms have been extensively introduced into the design of MEMS acoustic sensing systems, promoting their practicality and applicability in the collection of health information. In addition, MEMS accelerometers [71] and resonators [143] have also been introduced to form integrated MEMS systems for wearable or biomedical applications, together with acoustic sensors, providing more comprehensive and in-depth sensing methods.

## 7. Conclusions

In this article, we discussed the recent development of MEMS acoustic sensors, with a particular focus on four types of devices: piezoelectric, piezoresistive, capacitive, and optical. Regarding piezoelectric devices, ZnO and AlN materials have gained greater popularity and acceptance among researchers compared to PZT thin films, leading to their widespread application in various piezoelectric acoustic sensors. These sensors find applications in areas such as underwater acoustic detection, electronic stethoscopes, and wearable health monitoring. Among piezoresistive devices, piezoresistive MEMS hydrophones with biomimetic structures have been the focus in recent years. Whether emulating cilia or fish lateral lines, these devices offer considerable flexibility in designing geometric structures, allowing for the creation of various vector hydrophones. Capacitive devices, given their technological maturity, have been extensively commercialized and are popular in markets for consumer electronics. Additionally, capacitive MEMS acoustic sensors have been integrated into wearable health monitoring systems and have inspired biomimetic device structures that emulate animal eardrums. In terms of optical devices, grating interferometers, Fabry–Perot, and micro-opto-mechanics remain the preferred readout methods, known for their exceptional accuracy and sensitivity. Aside from the innovations in device structures and performance enhancement, researchers have developed several modeling methods and simulation strategies for piezoelectric, piezoresistive, and capacitive MEMS acoustic sensors. These approaches provide practical tools for designing key structures and predicting device performance.

Overall, MEMS acoustic sensors have undergone rapid development in recent years. With ongoing advancements in materials and analytical techniques, it is anticipated that future MEMS acoustic sensors will achieve improved performance and find widespread applications in consumer electronics, health monitoring, and underwater acoustic detection.

## Figures and Tables

**Table 1:** Performance comparison of several recent MEMS acoustic sensors based on different piezoelectric materials.

Ref.	Materials	Sensitivity	Frequency Bandwidth	Noise Resolution
[38]	PZT	11 mV/Pa	40 kHz–48 kHz	/
[39]		9.2 V/g	<1000 Hz	1 μV/√Hz
[29]	ZnO	80 μV/Pa	22 kHz	/
[50]		130 μV/Pa	48 Hz–54 kHz	/
[22]	AlN	4.49 mV/Pa (re. 1 V)	2 kHz–54 kHz	67.03 dB@1 kHz
[58]		−178 dB (re. 1 V/μPa)	100 Hz–1600 Hz	52.6 dB@100 Hz
[61]	ScAlN	−175 dB (re. 1 V/μPa)	20 kHz–160 kHz	/
[62]		−37.6 dB (re. 1 V/Pa)	100 Hz–20 kHz	40 dB

**Table 2:** Performance comparison of several recent piezoelectric MEMS hydrophones.

Ref.	Sensitivity	Frequency Bandwidth	Noise Resolution
[62]	−37.6 dB (re. 1 V/Pa)	100 Hz–20 kHz	40 dB
[65]	−180 dB (re. 1 V/μPa)	10 Hz–8 kHz	60 dB (re. 1 μPa/√Hz)
[66]	−178 dB (re. 1 V/μ Pa)	10 Hz–50 kHz	58.7 dB (re. 1 μPa/√Hz)
[67]	−189.3 dB (re. 1 V/μPa)	20 Hz–2000 Hz	/
[68]	−163 dB	20 kHz–200 kHz	/

**Table 3:** Performance comparison of recent bionic MEMS vector hydrophones based on ciliary structures.

Ref.	Features	Sensitivity	Frequency Bandwidth
[85]	CCVH	−183.3 dB@1600 Hz (re. 1 V/μPa)	20 Hz–1 kHz
[86]	DCVH	−186.1 dB@1 kHz (re. 1 V/μPa)	20 Hz–1 kHz
[87]	HCVH	−185.6 dB@1250 Hz (re. 1 V/µPa)	20 Hz–1 kHz
[88]	BCVH	−183.3 dB@1 kHz (re. 1 V/µPa)	20 Hz–1 kHz
[89]	CSCVH	−182.7 dB@1 kHz (re. 1 V/µPa)	20 Hz–1 kHz (linear bandwidth 20 Hz–678 Hz)
[90]	SCVH	−184.2 dB@1 kHz (re. 1 V/μPa)	20 Hz–1 kHz
[91]	CCCVH	−184.5 dB@1250 Hz (re. 1 V/μPa)	20 Hz–1250 Hz
[92]	3DCVH	−189 dB@500 Hz (re. 1 V/μPa)	20 Hz–500 Hz
[93]	FUVH	−188.5 dB@630 Hz (re. 1 V/μPa)	20 Hz–500 Hz
[94]	AFUVH	−177.53 dB@1 kHz (re. 1 V/μPa)	20 Hz–1 kHz
[95]	FUVH	−167.93 dB@1 kHz (re. 1 V/μPa)	20 Hz–1200 Hz

**Table 4:** Performance comparison of several recent capacitive MEMS acoustic sensors with different features.

Ref.	Features	Sensitivity	Frequency Bandwidth	Noise Resolution
[85,98]	Levitation-based electrode configuration	16.1 mV/Pa@1 kHz with a 200 V bias voltage	100 Hz–4.9 kHz	/
[100]	Cantilever-enhanced photoacoustic spectroscopy	3749 mV/Pa@1870 Hz with a 15 V bias voltage	17 Hz–1870 Hz	7.9 μPa/√Hz
[105]	Anti-stiction-dimple array	12.63 mV/Pa@1 kHz	4 Hz–4 kHz	−93 dBV
[106]	Backplate with center and peripheral protrusions	−38 dB@1 kHz	Resonant frequency: 39 kHz	/
[111]	*Ormia ochracea* tympana-inspired	−84.6 dB (re. 1 V/μPa) (59 V/Pa)	120 Hz–3 kHz	Maximum SNR of 88 dB

**Table 5:** Performance comparison of recent optical MEMS acoustic sensors based on different methods.

Ref.	Features	Sensitivity	Frequency Bandwidth	Noise Resolution
[85,118]	Grating interferometer	−15.14 dB@1 kHz	100 Hz–2.5 kHz	/
[119]	Grating interferometer	0.776 V/Pa@1 kHz	2.5 Hz–3.4 kHz	/
[121]	Fabry–Perot	950 nm/Pa	20 Hz–13 kHz	25.68 μPa/√Hz
[20]	Fabry–Perot	1.753 μm/Pa@1 kHz	/	0.21 μPa/√Hz
[21]	Fabry–Perot	2.6 V/Pa	100 Hz–18 kHz	230 μPa/√Hz
[123]	Micro-opto-mechanical	8.34 V/Pa	/	35 nPa/√Hz
[124]	Micro-opto-mechanical	0.1 V/Pa	50 Hz–18 kHz	3 μPa/√Hz

## Data Availability

Data are contained within the paper.
